# Supplementary material for: Interpretable candidate drug prioritization and explanation framework across-medical knowledge graphs based on graph embedding models: A case study of type 2 diabetes
Source: PLoS One. 2026 May 13;21(5):e0349026. doi: 10.1371/journal.pone.0349026 (PMC13170841; doi:10.1371/journal.pone.0349026)
Supplement: Attachment — Submitted filename: Response to Reviewers.docx [file pone.0349026.s002.docx]

**Manuscript ID:** PONE-D-25-64588

**Title:** Interpretable Drug Discovery Across Medical Knowledge Graphs Based on Graph Embedding Models: A Case Study of Type 2 Diabetes

Dear Academic Editor and Reviewers,

We would like to express our sincere gratitude to you and the reviewers for the thoughtful review and highly constructive comments regarding our manuscript. The feedback has been invaluable in helping us refine the logical structure, clarify our methodological contributions, and improve the overall rigor of our study.We have carefully studied all the comments and have made comprehensive revisions to the manuscript. All modifications have been highlighted in the **'Revised Manuscript with Track Changes'** file. Below, we provide a point-by-point response to the concerns raised by the Academic Editor and the Reviewers.

### **Part I: Response to Journal Requirements**

**Point 1:** Ensure manuscript meets PLOS ONE's style requirements.

**Response:** We have carefully checked and reformatted the manuscript according to the PLOS ONE style templates, including the title page, author affiliations, and file naming conventions.

**Point 2:** ORCID iD for the corresponding author.

**Response:** The corresponding author has successfully linked and validated their ORCID iD in the Editorial Manager system.

**Point 3 & 5 & 6:** Code sharing, Data Availability Statement (DAS), and Open Data Policy.

**Response:** We fully agree with PLOS ONE’s open science policy. We have now uploaded the complete dataset (including the fully processed 7 million+ triples of the cross-medical knowledge graph) and the source code (including AnyBURL rule learning and ComplEx link prediction) to a public repository.

- The Data Availability Statement has been updated in the submission system.
- **Access Link:**https://github.com/kunkunabc/T2DM-CrossMed-KG/tree/main

**Point 4 & 7:** Tables and Supplementary Figures format.

**Response:** All tables have been moved into the main text immediately following their first mention. The file types for supplementary figures have been correctly amended to **'Supporting Information'**, and their legends have been placed after the reference list in the manuscript.

### **Part II: Response to Reviewer #1**

**Reviewer 1, Comment 1:** Substantiation of Novelty: Dataset and Methodological Contribution. The manuscript currently overstates its novelty... The description suggests the authors' work primarily involves extending SymMap... Provide a transparent comparison. Reframe the contribution... Clarify what is new about the unified path scoring scheme.

**Response:** We deeply appreciate the reviewer's sharp observation. We agree that our previous narrative did not accurately reflect our actual contribution. We have made the following major revisions:

1. **Reframing the Narrative & Data Contribution:** We have thoroughly rewritten the Abstract and Introduction to explicitly state that our contribution is not creating a generic cross-medicine graph, but rather constructing a "domain-specific, scenario-driven knowledge graph tailored for T2DM". To provide a transparent comparison, we added a statistical analysis of the data sources in **Section 3.2.2.** The results show that while SymMap (32.51%) and TCMBank (13.03%) provide the backbone, we introduced Hetionet (29.72%) and STRING (23.81%) to significantly expand the biological depth. The non-TCM mechanistic data accounts for over 50% of the graph, effectively filling the gap in molecular pathway connectivity prevalent in traditional TCM databases.
2. **Methodological Advancement:** In **Section 4.3** (Unified Path Scoring Mechanism), we have explicitly articulated the novelty of our scoring scheme. Unlike traditional path-finding, we innovatively introduced an Ingredient Specificity Index (ISI) inspired by TF-IDF to filter out high-frequency generic noise (e.g., palmitic acid). Furthermore, we designed a Hybrid Path Confidence Calibration ($\lambda_{DFS}$) mechanism to solve the confidence space mismatch between deterministic DFS paths and probabilistic AnyBURL rules.

**Reviewer 1, Comment 2:** Mandatory Compliance with PLOS ONE Data Availability Policy.

**Response:** We apologize for the previous omission. As detailed in our response to the Journal Requirements, the entire processed knowledge graph and the source code have now been made fully available without restriction at https://github.com/kunkunabc/T2DM-CrossMed-KG/tree/main.

**Reviewer 1, Comment 3:** Experimental Rigor: Justify the choice of embedding models and evaluation metrics more thoroughly.

**Response:** We thank the reviewer for this suggestion. To rigorously justify our choice of the ComplEx model, we have added two classical baselines (TransE and DistMult) to our experiments. In **Section 5.1**, we provide a detailed comparative analysis, demonstrating that classical translational/bilinear models suffer from severe semantic expressional conflicts or loss oscillation when handling the massive "many-to-many" and asymmetric mappings in our graph, whereas complex space models (ComplEx) bypass these constraints perfectly.

**Reviewer 1, Comment 4:** Figure Clarity: Ensure all figures (especially Fig. 1 and Fig. 2) are high-resolution, legible, and contain self-explanatory legends.

**Response:** We have regenerated Fig. 1 and Fig. 2 with higher resolution and updated their legends to be completely self-explanatory.

**Reviewer 1, Comment 5:** Discussion of Limitations: Expand the limitations section...

**Response:** We have expanded the limitations in **Section 6 (Conclusion).** We explicitly discuss our dependency on the quality and coverage of existing databases (e.g., SymMap, STRING), potential data missingness issues, and the subjective *a priori* nature of the hyperparameter settings in our scoring scheme.

### **Part III: Response to Reviewer #2**

**Reviewer 2, Comment 1:** The motivation in both the abstract and the introduction should be strengthened... shift from merely stating what has been done to clearly articulating what problem is being addressed and solved.

**Response:** We completely agree. We have comprehensively rewritten the Abstract and the Introduction. The revised text explicitly articulates the core problem we are solving: generic integrative graphs lack the mechanistic resolution needed for specific complex diseases, and high-performing deep learning models act as "black boxes" that fail to provide traceable molecular cascades for clinicians. Our "scenario-driven" graph construction and unified scoring mechanism are directly proposed to solve this dual challenge.

**Reviewer 2, Comment 2:** Regarding related works, many recently published papers... were missed (10.1109/JBHI.2025.3600045, 10.1109/JBHI.2025.3585290, and 10.1109/JBHI.2024.3383591).

**Response:** We thank the reviewer for pointing out these highly relevant and recent publications. We have carefully reviewed these papers and incorporated them into the **Section 2 (Related Work)** to provide a more comprehensive and up-to-date literature review on advanced graph learning for complex disease drug discovery.

**Reviewer 2, Comment 3:** Include an overview figure illustrating the main workflow of the proposed framework.

**Response:** This is an excellent suggestion. We have added a new figure **(Fig. 1)** and a corresponding "Overall Workflow" subsection at the beginning of **Section 3**, which visually and textually highlights the four core stages of our framework: Data Integration, Gene-Bridged KG Construction, Link Prediction, and Interpretable Reasoning.

**Reviewer 2, Comment 4:** Methodological descriptions and experimental results should be presented separately to improve clarity and readability.

**Response:** We have fully restructured the manuscript according to this advice. The previous combined section has been cleanly split into **Section 4: Methodology Implementation (detailing embedding models, experimental settings, and the scoring mechanism)** and **Section 5: Results and Discussion (focusing purely on link prediction performance, candidate prediction, and case studies).**

**Reviewer 2, Comment 5:** Regarding the weighting scheme and hyperparameter settings... It is recommended to conduct lightweight sensitivity analyses.

**Response:** We appreciate this constructive feedback. In the newly added **Section 5.3.2 (Parameter Sensitivity Analysis)**, we conducted robust sensitivity experiments. We evaluated the Rank Consistency (IoU) of the Top-10 paths under different settings, proving that our framework achieves optimal balance when the DFS penalty factor ($\lambda_{DFS}$) is set to 0.2, and demonstrating that the model maintains strong global robustness without parameter overfitting.

**Reviewer 2, Comment 6:** Add comparative experiments to justify the selection of ComplEx and RotatE... including additional representative baselines.

**Response:** We have added TransE (translational distance model) and DistMult (bilinear diagonal model) as representative baselines. The new comparative results in **Section 5.1** clearly justify the superiority of complex space models in our specific cross-medicine heterogeneous graph context.

**Reviewer 2, Comment 7:** Ablation studies are necessary to demonstrate the necessity and contribution of each major design component.

**Response:** Thank you for this crucial suggestion. We have added a comprehensive Ablation Study (**Section 5.4**). By evaluating four variants (w/o ISI, w/o Weights, w/o Rules, and w/o Bonus) against metrics like Average Path Specificity and Evidence Ratio, we quantitatively demonstrated that our unified scoring framework is a tightly coupled organic whole, not a mere accumulation of discrete factors.

**Reviewer 2, Comment 8:** For future works, authors should discuss the possibility of applying advanced graph learning techniques (10.1038/s41551-024-01312-5 and 10.1109/TSMC.2025.3572738).

**Response:** We have updated the **Section 6 (Conclusion)** to discuss future directions. We explicitly cited the suggested papers, noting that introducing Transformer-based graph representation learning architectures and link-based attributed graph clustering algorithms could significantly enhance the model's ability to excavate high-order topological features and dynamically complete the graph.

Once again, we thank the Editor and Reviewers for their time, expertise, and highly constructive feedback, which have significantly elevated the quality of our manuscript. We hope that the revisions meet with your approval.

Sincerely,

Zekun Zhou

Corresponding Author:Shuo Yang

Affiliation:Institute of Information on Traditional Chinese Medicine, China Academy of Chinese Medical Sciences
