# Supplementary material for: Interpretable candidate drug prioritization and explanation framework across-medical knowledge graphs based on graph embedding models: A case study of type 2 diabetes
Source: PLoS One. 2026 May 13;21(5):e0349026. doi: 10.1371/journal.pone.0349026 (PMC13170841; doi:10.1371/journal.pone.0349026)
Supplement: Attachment — Submitted filename: Response_to_Reviewers_auresp_2.docx [file pone.0349026.s003.docx]

**Manuscript ID:** PONE-D-25-64588

**Title:** Interpretable Candidate Drug Prioritization and Explanation Framework Across-Medical Knowledge Graphs Based on Graph Embedding Models: A Case Study of Type 2 Diabetes

Dear Academic Editor and Reviewers,

We would like to express our sincere gratitude for your thorough and constructive evaluation of our manuscript (Manuscript ID: PONE-D-25-64588). We are particularly grateful for the insightful comments from Reviewer 3, which have significantly guided us in improving the rigor, interpretability, and presentation of our work. We have carefully addressed all the concerns raised. All modifications have been highlighted in the **'Revised Manuscript with Track Changes'** file. Below, we provide a point-by-point response to the reviewers' comments.

### **Response to Reviewer #1 and Reviewer #2:**

We sincerely thank you for your positive feedback and for endorsing the publication of our work. Your recognition of our unified path scoring framework and the cross-medical knowledge graph is highly encouraging.

**Response to Reviewer #3:**

**Comment 1: The prediction and validation design is not rigorous enough to support the drug discovery claims...**

**Response:** We thank the reviewer for this insightful comment. We have taken measures to ensure the rigor of our experimental design. Regarding the concern about potential data leakage, we wish to clarify that our link prediction was implemented using the PyKEEN framework, which strictly adheres to the 'Filtered' evaluation protocol. This protocol ensures that all known true triples in the graph are filtered out from the ranking candidates during testing, thereby preventing the model from simply 'recovering' information already encoded in the graph. We have added this technical clarification and the corresponding citation to **Section 4.2.**

**Comment 2: The conclusions overstate what the current evidence supports...**

**Response:** We thank the reviewer for this insightful suggestion. We agree that the term "drug discovery" may overstate our current evidence. Accordingly, we have substantially toned down our claims throughout the manuscript:

- We have revised the title, abstract, conclusion and the relevant sections in the main text to redefine our framework as an "interpretable candidate drug prioritization and explanation framework" rather than a discovery platform.
- We have repositioned the study as a hypothesis-generation tool designed to assist experts in prioritizing candidates for further experimental validation.
- We have explicitly added a new "Limitations and Future Work" section (**Section 5.6**) in the Discussion chapter, where we acknowledge the lack of direct experimental validation and discuss the inherent constraints of the current datasets.

**Comment 3: The interpretability framework is largely heuristic and is not itself validated...**

**Response:** We sincerely appreciate the reviewer's rigorous standard for the empirical justification of our scoring framework and the biological relevance of the identified paths:

1. **Empirical Justification via Ablation and Sensitivity Analysis.**

We would like to clarify that, in response to the constructive feedback from the previous round of review, we had already incorporated a **Parameter Sensitivity Analysis (Section 5.3)** and an **Ablation Study (Section 5.4)** into the manuscript. As shown in **Section 5.3** and **Section 5.4**, the manual weights and the confidence calibration factor $\lambda_{DFS}$ are essential for maintaining the specificity of the ranking. Our results confirm that these heuristic constraints effectively suppress generic high-degree nodes, ensuring that the prioritized paths remain biologically relevant.

2. **Clarification on Mechanistic Paths: Regarding the reviewer's concern about the specific nodes.**

- **"Irritability":** We would like to point out that the path involving "irritability" had already been filtered out in our previous revision of the manuscript (the second version) as it did not meet our refined specificity threshold.
- **"Bipolar Disorder" (Table 10, Path 7):** We have retained this path because it represents a well-documented neuro-metabolic crosstalk mechanism for Topiramate. As supported by literature [45,46], modulating pathways related to bipolar disorder can indirectly influence energy metabolism and insulin sensitivity through the regulation of PVALB. We have further refined the discussion in **Section 5.5** to clarify that this is a secondary regulatory mechanism, reflecting the complex systemic nature of antidiabetic therapeutics.

**Comment 4: The manuscript contains a major internal inconsistency in the model comparison.**

**Response:** We sincerely apologize for any confusion caused by the clerical inconsistency in the original version of our manuscript. We wish to clarify that in the current revised version, we have meticulously synchronized the results across the Abstract, Results, and Conclusion sections. We confirm that ComplEx consistently outperformed all other baseline models, and this conclusion is now accurately reflected throughout the entire paper.

**Comment 5: There is insufficient rigor in the quantitative evaluation.**

**Response:** We sincerely appreciate the reviewer's professional suggestion regarding the statistical rigor of our quantitative evaluation. To address this, we have systematically re-evaluated all four models using three different random seeds (42, 123, and 2026) to assess their stability.

Following your advice, we have updated Table 6 to report the primary link prediction metrics (MRR and Hits@K) as Mean ± Standard Deviation (SD). To manage the immense computational overhead of our 7.1-million-triple graph while strictly guaranteeing a fair comparison, these stability tests were conducted under identical parameters for 30 epochs. As shown in the updated table, the extremely low standard deviations (e.g., SD±0.003 for ComplEx Hits@10) empirically validate that the superior performance of the ComplEx model is statistically significant and not due to stochastic initialization.

Furthermore, to ensure absolute clarity regarding our methodology, we have refined **Section 4.2 (Experimental Settings)** and **Section 5.1（Link Prediction Performance）** to detail our two-stage evaluation strategy:We utilized the multi-seed tests (30 epochs) as rigorous statistical validation to establish model stability and relative ranking.Once the superiority of ComplEx was empirically proven, the final downstream candidate prioritization was performed using the fully converged ComplEx model (trained for 150 epochs) to maximize predictive sensitivity and biological accuracy.

We believe this approach fulfills both the rigorous statistical requirements of model comparison and the practical need for high-precision biological discovery.

**Comment 6: Entity alignment and relation fusion need validation.**

**Response:** We appreciate the reviewer’s constructive comments regarding the validation of our graph construction. Given the massive scale of the CMKG (over 7.1 million triples), exhaustive manual verification is unfeasible. Therefore, we adopted a stratified random sampling strategy to evaluate the most representative and cross-systemically integrated components of the graph.

1. **Rationale and Methodology for Validation.**

To ensure the high-fidelity integration of Traditional Chinese Medicine (TCM) and Modern Medicine（MM）, we selected Genes, Diseases, and Herbs as the target domains for validation. These entities serve as the primary semantic bridges in our graph and possess the widest coverage across the five source databases (Hetionet, SymMap, TCMBank, STRING, and TTD). By validating these high-degree nodes, we can robustly infer the reliability of the overall graph structure.

2. **Quantitative Results of Entity Alignment.**

We manually audited 100 randomly sampled entities (40 Genes, 30 Diseases, and	30 Herbs). By tracing each entity back to its original database identifiers and	biological definitions, we observed an Alignment Accuracy of 98.0%. This	confirms that our name-based reassignment protocol effectively maintains	semantic consistency across heterogeneous sources.

3. **Validation of Relation Fusion Logic.**

To test the efficacy of our statistical thresholds (Jaccard ≥ 0.8 or Jaccard ≤ 0.2 ,	Overlap ≥ 0.6), we audited 60 triples from the merged and filtered subsets:

- **Accuracy of Merged Relations (93.3%):** 28 out of 30 triples (primarily Anatomy-Gene pairs) were biologically rational. The minor errors (6.7%) highlighted a need for future Entity-Type Constraints to prevent compounds/diseases from inheriting tissue-specific predicates.
- **Accuracy of Evidence Filtering (86.7%):** 26 out of 30 triples were correctly kept separate to preserve evidence granularity. We identified 4 false negatives where physical bindings (e.g., CALM1-PDE1A) were statistically underestimated by source databases.

**Comment 7: Data and code availability are not yet adequate for reproducibility.**

**Response:** We fully agree with the importance of reproducibility. We apologize for the confusion, which may have stemmed from the asynchronous nature of the previous revision cycle.While a repository was established in the previous revision, we have significantly updated it to include new resources generated in response to your specific feedback (Comments 5 and 6).The updated repository is available at:**https://github.com/kunkunabc/T2DM-CrossMed-KG/tree/main.**

**Key additions to the repository include:**

- **Statistical Rigor (Comment 5):** Complete scripts and raw results for multi-seed training (seeds 42, 123, 2026) across all models to ensure performance stability.
- **Graph Validation (Comment 6):** Stratified sampling scripts and the manual audit tables (98.0% entity accuracy and 93.3% relation rationality) providing the ground-truth verification requested.

We have updated the Data Availability Statement in the manuscript to include this link, ensuring all data and code are fully accessible for peer review and future research.

**Comment 8: The writing requires substantial editing before publication.**

**Response:** We apologize for the grammatical and stylistic errors. We have conducted a comprehensive linguistic proofreading of the entire manuscript. Specifically:

- We have corrected the identified typos, such as changing "Traditional chinese medicine" to "Traditional Chinese Medicine," and "burnden" to "burden."
- We have rectified inconsistent capitalization (e.g., "Mediacal Insurance") and punctuation errors (e.g., missing spaces after periods).
- We have standardized the use of terminology and improved the sentence structures for better clarity and academic rigor.
- In addition, we further checked other grammatical expressions and other issues throughout the text and highlighted them in the **Revised Manuscript with Track Changes**.

We hope that these revisions adequately address your concerns and make the manuscript suitable for publication.

Sincerely,

Zekun Zhou

Corresponding Author:Shuo Yang

Affiliation:Institute of Information on Traditional Chinese Medicine, China Academy of Chinese Medical Sciences
